# 60 Minutes Per Day in Moderate to Vigorous Physical Activity as a Natural Health Protector in Young Population

**DOI:** 10.3390/ijerph17238918

**Published:** 2020-11-30

**Authors:** Joanna Baran, Aneta Weres, Justyna Wyszyńska, Grzegorz Pitucha, Ewelina Czenczek-Lewandowska, Wojciech Rusek, Justyna Leszczak, Artur Mazur

**Affiliations:** 1 Institute of Health Sciences, Medical College, University of Rzeszów, Al. mjr.W.Kopisto 2a, 35-310 Rzeszów, Poland; anetaweres.ur@gmail.com (A.W.); justyna.wyszynska@onet.pl (J.W.); e.czenczek@univ.rzeszow.pl (E.C.-L.); leszczakjustyna.ur@gmail.com (J.L.); 2 Institute of Agricultural Sciences, Protection and Management of Environment, College of Natural Sciences, University of Rzeszów, ul. Ćwiklińskiej 1a, 35-601 Rzeszów, Poland; gpitucha@wp.pl; 3 REHAMED-CENTER Sp. z o.o. Rehabilitation Center, Tajęcina 66a, 36-022 Tajęcina, Poland; rusekaw@interia.pl; 4 Institute of Medical Sciences, Medical College, University of Rzeszów, Al. mjr.W.Kopisto 2a, 35-310 Rzeszów, Poland; drmazur@poczta.onet.pl

**Keywords:** children, exercise, obesity, sport, weight disorders, young people

## Abstract

The aim of this study was to check whether following the recommendations of the World Health Organization (WHO) concerning physical activity protects children and adolescents against the occurrence of overweight and obesity. A total of 1002 children were included in the analysis. The average age of the studied children was 9.36 years ± 3.52 years. Measurement of height and body mass was performed for every child, followed by physical activity assessment over seven days. In each group analyzed, the overweight and obese children had a shorter time in moderate to vigorous physical activity (MVPA) than the children with normal body mass. Among the children spending < 60 min per day in MVPA, the risk of occurrence of overweight and obesity was greater than among children with physical activity > 60 min per day. It was also shown that the greatest risk of occurrence of body mass disorder was a situation in which the mother’s gestational weight gain (GWG) is over 18 kg and the child does not spend a minimum of 60 min/day in MVPA. Not meeting the recommendation is connected with an increased chance of the occurrence of overweight and obesity. The level of physical activity influences the incidence of overweight and obesity. The children with overweight and obesity spent less time per day in MVPA. An increased body mass in mothers during pregnancy associated with a child’s low level of physical activity increases the risk of the occurrence of overweight and obesity.

## 1. Introduction

Physical activity is any movement made by the human body using the skeletal muscles. It is of great importance as a preventive and healing factor [1]. The developmental period is characterized by large changes in the child’s body, especially in the field of the musculoskeletal and cardiovascular system, motor coordination and movement control. A large role is assigned to shaping body composition, which can be regulated through physical activity [2]. Exercise reduces sympathetic activation and blood pressure, improve insulin sensitivity, lipid profile, as well as promote the expenditure of excess energy from adipose tissue while reducing body weight [3]. Children who are overweight or obese are much more likely to develop further noncommunicable diseases in adulthood, including insulin resistance (pre-diabetes), diabetes, arterial hypertension, coronary artery disease, and fatty liver disease. Therefore, the implementation of regular physical activity as a permanent habit in the lifestyle of overweight and obese children, as well as the correct bodyweight, is crucial for their health [4].

In order for physical activity to play the role of a preventive factor, it must be performed frequently, regularly and with a given intensity. It is commonly accepted that children and adolescents aged 5–17 (regardless of sex, race or social status) should spend at least 60 min per day in moderate to vigorous physical activity (MVPA), e.g., fun and games, sport, housework, recreation, physical education, or planned exercises in the context of family and school activities. Physical activity over 60 min provides additional health benefits [5]. Most of the daily physical activity should be aerobic. Intensive exercises, including those that strengthen muscles and bones (compression, stretching, torsional movements), should also be included at least three times per week [6]. In addition, it should be remembered that obesity does not appear in a child within a short time and often, its causes go back to the prenatal period. Therefore, it is important to take into account also factors that may affect the baby during pregnancy [7,8,9]. In the available literature, there are no connections about, i.e., the influence of maternal weight gain during pregnancy and the current level of physical activity of the child on the odds of obesity in this child. There is also little data on the study of physical activity of children with weight disorders using accelerometers. There are many more publications in which the authors used simpler devices—pedometers, while a 3-axis accelerator is a safe method of assessing activity in adults, children and pregnant women. Unlike pedometers, they record the user’s movement in all three axes, thanks to which it is possible to distinguish between real movement and, e.g., walking on the spot or moving a hand [10,11,12,13,14].

There is a great need to analyze physical activity in the context of a preventive factor in the development of overweight and obesity in children, as well as to constantly monitor the level of compliance with the standards of physical activity [15]. This is necessary to detect excessive weight gain in some children quickly enough, enabling early interventions to be implemented. Hence the aim of the study was to check whether meeting the World Health Organization (WHO) recommendation on physical activity protects children and adolescents against becoming overweight or obese. Moreover, our second goal was to check the influence of a child’s current level of physical activity and the maternal weight gain during pregnancy on the odds of obesity in this child due to the lack of the above data in the available literature.

## 2. Materials and Methods

### 2.1. Participants

The study covered 1300 school and kindergarten children. The final size of the study group was affected by the specified inclusion criteria (consent of the parent or legal guardian and the child to participate in the study, age of the child between 3 and 18 years old) and the exclusion criteria (disability or injuries in the lower limbs preventing physical activity).

After the parents’ written consent was obtained, 1196 children were qualified for the study and asked to wear the accelerometer. Of those, 74 had insufficient recording time on weekdays, 86 had insufficient recording time on free days (Saturday-Sunday), and 34 did not achieve a sufficient number on weekdays and weekends. Finally, 1002 children were included in the analysis.

The average age of the studied children was 9.36 ± 3.52 years. In the studied group, there was a slightly higher number of boys compared to girls (527 vs. 475).

The research was based on a group of children, as children’s young bodies are constantly developing. Physical activity is a modifiable factor that can protect against excessive body weight [16], diabetes [17], arterial hypertension [18], posture defects [19] in children or depression [20].

Additionally, the study group was divided into age groups. This division is based on the different stages of children’s motor development as well as the different motor needs of the child. Children aged 4–6 show a greater tendency to mobility and a greater natural need for exercise. Older children are less likely to exercise, especially in adolescence.

In addition, the project collected information about the children’s parents and perinatal factors.

### 2.2. Methods

Schools participating in the study were selected by random sampling. Each participant was examined while on an empty stomach, and subsequent measurements were taken in the morning hours. Measurements of body height and weight were performed for each child, followed by physical activity assessment over 7 days.

In addition, parents completed a questionnaire providing basic information about the child and family and provided a photocopy of the child’s health book and mother’s pregnancy card.

The research was approved by the Bioethics Committee of the University of Rzeszów No. 18/12/2015 of 2 December 2015.

### 2.3. Anthropometric Measurements

Body height was measured using a Seca 213 stadiometer (Seca, Hamburg, Germany). The subject stood barefoot, with his back to the measuring part of the height meter. Body height was measured 3 times, and the mean value was calculated to eliminate measurement error.

Bodyweight was assessed using a Tanita BC 420 MA analyzer (Tanita, Tokyo, Japan). The subject stood barefoot on the analyzer. The upper limbs were placed along the torso, held slightly away from it. The tester entered the age and body height of the subject into the analyzer software. After about 10 s, the result was obtained in the form of a printout.

### 2.4. Physical Activity Assessment

Physical activity was assessed using the accelerometer Actigraph wGT3X-BT monitor (Actigraph, Pensacola, FL, USA). Each participant wore an accelerometer on the right side, above the hip, for 7 consecutive days of the week from morning to evening (put on immediately after waking up and taken off immediately before going to sleep). The children were required to wear the accelerometer during all physical activities (e.g., sports, dance, recreational, physical education, etc.). The accelerometer was removed only for sleep, during a visit to the swimming pool and for bathing (the device is not waterproof).

Valid days of physical activity, subject to further evaluation, were considered for the results covering a minimum of 4 days of the recording material (at least 500 min a day), including a minimum of 3 weekdays and a minimum of 1 day of the weekend. The remaining results were considered invalid and rejected [21]. The level of physical activity was determined according to research conducted by Evenson et al. [22], which identified the following division of levels of physical activity:

- sedentary: 0–100 counts per minute (CPM);
- light: 101–2295 CPM:
- moderate: 2296–4011 CPM;
- intensive: 4012–∞ CPM.

In addition, information on the increase in the mother’s weight during pregnancy was included in the analysis.

After all of the above data were collected, the children’s current body mass index (BMI) percentile was determined in relation to Polish centile grids [23], and the children’s body mass category was determined based on the BMI centile in relation to the classification by Barlow et al. [24].

### 2.5. Statistical Analysis

Data analysis was performed using selected methods of descriptive statistics and statistical inference. Selected numerical characteristics of the tested parameters were determined: arithmetic mean (x), median (Me), the highest (maximum) and lowest (minimum) values, standard deviation(s).

The verification of research hypotheses was carried out—depending on the nature of the compared features—by means of appropriate statistical tests. The level of statistical significance (p) was adopted where *p* < 0.05.

To assess the differences in percentage distribution (or the incidence of a certain phenomenon) in the compared groups, the chi-squared test of independence was used, which is a statistical test used to examine the relationship between two features measured on a nominal scale. Where the relationship between measurable and nominal characteristics was tested, then the Mann–Whitney test was used.

Odds ratio (OR) analyses of the probability of children being overweight or obese depending on specific parameters were also performed. The chance of occurrence of a given phenomenon is defined as the result of dividing the probability of its occurrence by the probability of the opposite event. In order to assess whether the difference in the incidence of a given phenomenon is statistically significant, a 95% confidence interval for OR is also given. If this range does not include the value of 1, then there may be a significant difference in the chance of occurrence of a given phenomenon in the two compared groups.

## 3. Results

Statistically significant differences were found showing higher rates of physical activity among boys. They showed longer time spent in MVPA (both in minutes *p* < 0.001, *p* = 0.001 and percentage *p* < 0.001, *p* = 0.003, respectively), longer time spent in MVPA (*p* < 0.001), higher average CPM (*p* = 0.001), higher average step counts (*p* = 0.006) and shorter sedentary time (*p* = 0.024) (Table 1).

The data on time spent in MVPA stratified by age and sex are shown in Table 2. When analyzing the MVPA distribution, attention should be paid to the median, not the mean, because the latter is exaggerated by a relatively small number of children achieving a very high level of daily physical activity.

There were differences in the level of activity relative to the child’s sex—especially in the oldest age group (*p* = 0.002). Boys aged 12–15 spent an average of 12.7 min more in moderate to vigorous physical activity compared to girls. Among the youngest children (4–6 years), the difference was also statistically significant (*p*= 0.016) and averaged 9.1 min in favor of boys.

Age significantly differentiates the level of girls’ activity (*p* = 0.037). In this case, it was the youngest and oldest groups that had the longest time spent in moderate to vigorous physical activity (Table 2).

Table 3 presents the median value of MVPA in relation to the dichotomous division into overweight (and obese) children and with BMI in the norm (or below), in groups divided according to sex and age (it was decided to present the median, due to the asymmetry of the MVPA distribution as it better reflects the average level of this variable). It was shown that the oldest girls who were overweight or obese spent significantly less time in MVPA than girls with normal body weight in this age group. (*p =* 0.041). An analogous result was obtained, taking into account the whole group of 12–15 year-olds (*p* = 0.024) and the entire study group (*p* = 0.049). In addition, in each of the analyzed groups, overweight and obese children had a shorter time in MVPA than children with normal body weight.

A detailed analysis of the occurrence of excessive body weight was also carried out, with a breakdown by age and sex. The results are presented in a simplified manner, presenting only the number and percentage share of overweight/obese subjects in the groups under comparison.

This analysis showed that in the oldest group of girls, excessive body weight occurred twice as often if the subject did not meet a minimum of 60 min per day in MVPA (*p =* 0.043) (Table 4.).

Among the children spending < 60 min per day in MVPA, the risk of overweight or obesity was greater than in the children with MVPA > 60 min per day (Table 5).

Due to numerous reports on the impact of maternal weight gain during pregnancy on the subsequent occurrence of childhood obesity, the incidence of overweight/obesity in the separate groups was compared in relation to the mother’s weight gain during pregnancy and the child’s current physical activity. The reference point was a group where mothers gained no more than 18 kg and children met the recommendation for physical activity. It has been shown that the greatest risk of weight disorders is when a pregnant mother gained over 18 kg and the child does not spend at least 60 min/day in MVPA (Table 6).

## 4. Discussion

Low levels of physical activity at a young age are associated with a number of adverse health effects. Many systematic reviews suggest the importance of promoting more physical activity, healthy diets and nutrition for good health-related quality of life among children and adolescents. [25,26] In addition, there is scientific evidence that sedentary behaviors during adolescence are negatively associated with adolescent health outcomes, such as obesity and the risk of metabolic diseases. Sedentary time can also have an influence on health states in adult ages. Increased body weight and reduced physical activity predispose to an increased risk of mental cardiovascular, motor or metabolic diseases [27,28,29].

Our research showed a number of significant results regarding the relationship between physical activity and body weight in the studied children. First, boys were more active than girls in terms of steps counts, MVPA and each of its components. Girls, on the other hand, showed a greater proportion of the daytime spent in sedentary activities.

Second, in both the girls and boys groups, in each age group, obese children spent less time in MVPA activities per day. This difference was particularly noticeable in the whole study group, the whole group of 12–15-year-old children and the group of 12–15-year-old girls. This was confirmed by statistical significance tests. This is also confirmed by the odds ratio (OR = 1.36 95% CI: 0.97–1.89. *p* = 0.033) of overweight or obesity in children who do not meet the WHO recommendations for 60 min a day in MVPA. Additionally, 2 times more girls aged 12–15 years who did not meet the standard of 60 min a day MVPA was in the group of obese children (in relation to children with normal body weight).

The last result obtained by us, complementing the above summary, was the greatest chance of overweight or obesity in children who do not meet the WHO recommendation for physical activity and in addition, their mothers gained over 18 kg during pregnancy, which is the maximum recommended weight gain and concerns women with underweight before pregnancy (women who are normal or overweight or obese should gain less weight correspondingly). This is a nearly 2.5 times greater chance of obesity in a child in relation to children who meet the 60 min a day MVPA standard and whose we have gained less than 18 kg during pregnancy [30].

The results of our research on the time spent in MVPA by obese and non-obese children are consistent with the results of other researchers. Compliance by children with the WHO recommendations on spending a minimum of 60 min a day in MVPA shows a highly protective effect against the occurrence of overweight or obesity [31]. This is confirmed by scientific evidence. Children who are overweight or obese spend significantly less time in MVPA in relation to children with normal body weight. This applies to both girls and boys [32].

What is more, girls and boys whose body weight was classified as normal or below normal spent 5–20 more time in moderate and vigorous physical activity than overweight, obese or very obese children. These data are consistent with our research results. Moreover, the largest disparities were noted in the age group of 6–8 years; at the same time, this age group was the most physically active among both girls and boys [33].

Children with normal body weight had the longest daily time in MVPA (on average 88 min vs. 45 min for overweight and obese children) [34] as well as MVPA during school time (16.6 min vs. 19.6 min), during weekdays outside of school (20.9 min vs. 25.5 min) and during the weekend (35.6 min vs. 46.5 min) [35].

Another issue analyzed was meeting or not to meet the 60 min a day recommendation at the MVPA. Meeting the minimum daily standard of 60 min in MVPA also turns out to be extremely important in protecting against the occurrence of overweight and obesity. According to Vale et al., boys more often achieved the recommendations of ≥1 h MVPA and ≥3 h TPA than girls. Noncompliance with the recommendations of ≥1 h MVPA was associated with obesity (OR = 3.8; IC: 1.3–10.4) only in girls [36], which was also confirmed in our research.

Diouf et al. also showed that in a group of children who meet the recommendations for spending a minimum of 60 min a day in MVPA, there were the most children with normal body weight and only below 10% of overweight and obese children [34]. These results are similar to the results of the author’s own research. Statistical analysis showed that in the case of girls and boys aged 7–11 years and 12–15 years, the greater percentage of children who are overweight or obese was in the group of children who did not meet the recommendations of 60 min daily in MVPA.

The latest analysis, supplementing the knowledge with maternal factors, also brought some interesting observations. It was also found that both excessive GWG and the level of physical activity of the child interact with each other increasing the risk of overweight and obesity in the child. Three models were compared: (1) GWG in the norm and the child’s physical activity < 60 min/day, (2) GWG above the norm and the child’s physical activity < 60 min/day, (3) GWG above the norm and the child’s physical activity > 60 min/day; in relation to GWG in the norm and the child’s physical activity > 60 min/day. It has been shown that each of the three models increased the risk of overweight and obesity in the child; however, the highest odds ratio is brought by the combination of GWG above the norm and low physical activity of the child (OR = 1.68; 95% CI: 0.88–3.18; *p* = 0.048).

Such results were also obtained by other researchers confirming the adverse effect of excessive GWG on the child’s body weight in the later years of his life. Higher maternal GWG was associated with a greater BMI of the offspring, and the risk of being overweight doubled in children whose mothers were overweight/obese before pregnancy and gained weight during pregnancy [37,38].

Insufficient GWG is associated with an increased risk of a child being underweight, while excessive GWG is associated with an increased risk of overweight and obesity [39]. Overweight/obesity of pregnant mothers and excessive GWG are associated with greater weight gain and increased length of offspring in early infancy also. Excessive GWG has been associated with an increased risk of overweight and obesity in infants [40]. Both high pre-pregnancy BMI and inappropriate GWG are associated with higher BMI in offspring. Pregnant women should try to achieve appropriate weight gain to prevent obesity in their children [41].

Despite the efforts, the authors did not avoid limitations in the design of the research. One of the weaknesses is the lack of information about parents from direct examination (the data only comes from interviews) and the lack of data on children < 4 years of age and >15 years of age, which would allow the presentation of results for the entire population of children from the studied region.

However, this study has many strengths, which include a relatively large study group (in general), comparable numbers of children by sex and place of residence, and the fact that measurements were taken on accelerometers, which are objective devices. The indicated features of the study group have a positive impact on the reliability of the tests.

The conducted research proved the beneficial effect of physical activity in protecting the child against overweight or obesity. However, bearing in mind the above limitations, some modifications should be introduced when planning further studies. The first is to plan longitudinal studies, starting from the prenatal period through the early childhood period to school age. This would undoubtedly have an influence on the extension of the time and cost of the research; however, it would provide valuable information, e.g., on the course of pregnancy, mother’s weight gain during pregnancy, body weight changes from the neonatal period to the school period, and all these data would come from a direct study.

In addition, when recruiting educational institutions, it would be worth qualifying a larger number of institutions so that the study also covers children < 4 years of age and> 15 years, which the authors did not manage to do in this study.

The take-home message from this research is that even small amounts of regular physical activity can protect against excessive weight. Maintaining appropriate weight gain during pregnancy and preventing overweight/obesity in mothers before pregnancy should also be a strategy for preventing overweight/obesity in children.

## 5. Conclusions

Failure to meet the standard is associated with a greater chance of overweight and obesity. The level of physical activity affects the incidence of overweight and obesity. Children who had > 60 min daily MVPA were more likely to have normal or below normal body weight. Overweight and obese children spent less time a day in MVPA. Among 12–15-year-old girls with activity < 60 min a day, the incidence of overweight and obesity was twice as high as in girls who meet the recommendations. Excessive weight gain of a pregnant mother, combined with a low level of physical activity of the child, increases the risk of overweight and obesity.

## Figures and Tables

**Table 1:** Characteristics of parameters describing the level of physical activity of the participants depending on sex.

Physical Activity Measure	Sex								*p*
	Girls				Boys				
	*N*	$\bar{x}$	Me	SD	*N*	$\bar{x}$	Me	SD	
Sedentary (min)	475	3329	3294	915	527	3264	3233	938	0.291
Light (min)	475	1073	1046	352	527	1105	1077	375	0.246
Moderate (min)	474	239	220	129	527	262	234	134	<0.001
Vigorous (min)	475	175	135	172	527	190	143	173	0.001
Sedentary (min/day)	475	539.7	534.4	109.9	527	528.2	521.2	113.8	0.087
Light (min/day)	475	174.1	174.1	49.2	527	178.8	175.1	51.3	0.265
Moderate (min/day)	475	38.4	34.9	18.1	527	42.5	38.6	20	<0.001
Vigorous (min/day)	475	27.8	20.9	24.7	527	30.7	23.8	26.6	0.002
Sedentary (%)	475	69.0	70.1	9.8	527	67.5	68.6	10.3	0.024
Light (%)	475	22.5	22.2	6.4	527	23.1	22.7	6.8	0.240
Moderate (%)	475	5.0	4.6	2.4	527	5.5	5.0	2.6	<0.001
Vigorous (%)	475	3.6	2.8	3.2	527	3.9	3.1	3.3	0.003
MVPA per day (min)	475	66.2	55.7	40.8	527	73.2	62.2	43.0	<0.001
CPM	475	562.9	497.7	315.1	527	609.1	536.8	338.6	0.001
Steps counts per day (thousands)	475	8.8	8.6	2.7	527	9.4	9.0	3.0	0.006
Time (min)	475	4817.3	4858.7	1020.4	527	4820.1	4898.2	1061.8	0.047

CPM—counts per minute, Me—median, MVPA—moderate to vigorous physical activity, *N*—number of participants, *p*—test probability value calculated using the Mann–Whitney test, SD—standard deviation $\bar{x}$—average.

**Table 2:** Time spent in moderate to vigorous physical activity (MVPA) by sex and age of participants.

Age Group	MVPA Per Day [min]								*p*
	Girls				Boys				
	N	$\bar{x}$	Me	*SD*	N	$\bar{x}$	Me	*SD*	
4–6 years	114	66.7	52.9	46.6	128	73.8	62.0	44.5	0.016
7–11 years	217	65.9	60.4	32.9	252	69.5	64.1	35.2	0.080
12–15 years	144	66.1	52.2	46.6	147	78.8	59.7	52.4	0.002
*p*	0.037				0.946				

Me—median, *N*—number of participants, *p*—test probability value calculated using the Mann–Whitney test, SD—standard deviation $\bar{x}$—average.

**Table 3:** The relationship between time spent in MVPA and bodyweight category (dichotomous division) taking into account the sex and age of the participants.

	Body Mass Category According to BMI Centile								
	Girls			Boys			ALL		
	Healthy Weight	Overweight/Obese	*p*	Healthy Weight	Overweight/Obese	*p*	Healthy Weight	Overweight/Obese	*p*
MVPA (min/day)									
All	57.0	52.2	0.160	62.5	59.7	0.160	60.4	55.1	0.049
4–6 years	59.3	52.8	0.351	71.0	61.6	0.670	65.9	57.0	0.206
7–11 years	60.9	58.4	0.617	64.8	59.4	0.086	62.7	58.9	0.104
12–15 years	54.6	45.0	0.041	60.4	57.3	0.423	58.2	52.0	0.024

*p*–test probability value calculated using the Mann–Whitney test.

**Table 4:** The relationship between the occurrence of overweight and obesity and the recommended time of physical activity, taking into account the sex and age of the participants.

Occurrence of Overweight and Obesity According to Age and Sex	Time of Daily Physical Activity				*p*
	<60 min		>60 min		
	*N*	%	*N*	%	
Girls 4–6 years	7	10.0%	6	14.0%	0.522
Girls 7–11 years	17	16.0%	16	14.4%	0.739
Girls 12–15 years	27	30.7%	9	15.8%	0.043
Boys 4–6 years	10	16.9%	13	19.4%	0.722
Boys 7–11 years	19	18.1%	16	11.0%	0.107
Boys 12–15 years	16	20.0%	13	17.6%	0.704

*N*—number of participants, *p*—test probability value calculated using the Mann–Whitney test, %—percent.

**Table 5:** Odds ratio of overweight and obesity depending on the time of daily MVPA.

	Time of Daily MVPA					*p*
	>60 min		<60 min			
	*N*	%	*N*	%	*OR*	
Occurrence of overweight and obesity	73	14.7%	96	18.9%	1.36 (0.97–1.89)	0.033

*N*—number of participants, OR—odds ratio, *p*—test probability value calculated using the Mann–Whitney test, %—percent.

**Table 6:** Odds ratio of overweight and obesity depending on the mother’s weight gain during pregnancy and the child’s level of physical activity.

Weight Increase of Mother in Pregnancy/Activity of the Child	Occurrence of Overweight/Obesity (*p* = 0.048)		All
	*N* (%)	*OR*	
Up to 18 kg/>60 min	32 (11.6%)	1	275
Up to 18 kg/<60 min	47 (16.2%)	1.47 (0.91–2.38)	290
Over 18 kg/>60 min	17 (18.1%)	1.68 (0.88–3.18)	94
Over 18 kg/<60 min	21 (23.3%)	2.31 (1.25–4.26)	90

*N*—number of participants, OR—odds ratio, *p*—test probability value calculated using the Mann–Whitney test, %—percent.
